# Elucidation of site-specific red-ox kinetics in the CO-assisted N_2_O decomposition over Fe–ferrierite by combining modulation excitation with *operando* EPR spectroscopy†

**DOI:** 10.1039/d4sc07195f

**Published:** 2025-02-04

**Authors:** Jörg W. A. Fischer, Filippo Buttignol, Alberto Garbujo, Davide Ferri, Gunnar Jeschke

**Affiliations:** a Department of Chemistry and Applied Biosciences, ETH Zurich CH-8093 Zurich Switzerland gunnar.jeschke@phys.chem.ethz.ch; b Paul Scherrer Institute, PSI Center for Energy and Environmental Sciences CH-5232 Villigen Switzerland davide.ferri@psi.ch; c Basic Research Department, Casale SA CH-6900 Lugano Switzerland

## Abstract

The catalytic conversion of N_2_O over Fe-exchanged zeolites is an essential process for controlling its anthropogenic emissions and detrimental environmental impact. In the present study, we monitored an industrial Fe–ferrierite catalyst under conditions of CO-assisted decomposition of N_2_O using *operando* electron paramagnetic resonance (EPR) spectroscopy within the modulated excitation (ME) paradigm. Exploiting this approach, we demonstrated that N_2_O decomposition occurs *via* reversible Fe^II^/Fe^III^ transitions localized exclusively on isolated Fe^II^ centers located in the β-cationic position, successfully distinguished among various spectator species. The temporal evolution of the reversible β-Fe^II^/Fe^III^ transitions under oxidizing and reducing atmospheres was determined with multivariate curve resolution (MCR) and *via* double integration of their EPR signal, allowing us to calculate the apparent activation energies for the oxidation and reduction half-cycles. Despite the reaction is controlled by the reduction half-cycle, *i.e.* N_2_O promotes full oxidation of the active β-Fe^II^ centres irrespective of temperature, the kinetic results indicate that temperature enhances the rate of this oxidation reaction more than the rate of reduction in CO-rich conditions. This study shows that quantitative and qualitative reaction monitoring at sub-minute temporal resolution *via operando* EPR spectroscopy is possible and sufficient signal-to-noise can be obtained if the experiments are performed according to the ME approach and if phase-sensitive detection (PSD) is employed. Furthermore, our results also indicate that analytical methods, such as MCR, can produce reliable results in the framework of time-resolved EPR spectroscopy.

## Abstract 2

MES-PSD aided *operando* EPR spectroscopy revealed that in CO-assisted N_2_O decomposition over Fe–ferrierite only isolated Fe^2+^ in β positions undergo reversible redox cycling. Rate constants and activation energies for each half-cycle were obtained.

## Graphical abstract

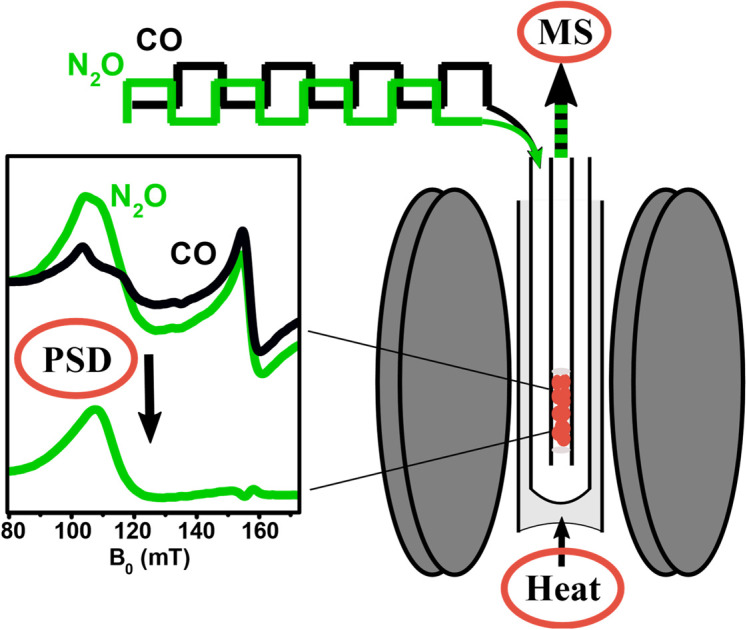

## Introduction

Electron paramagnetic resonance spectroscopy (EPR) is a powerful tool to gain insight into the nature and role of transition metal ions (TMIs) in catalytic processes.^1^ Due to its working principle, EPR spectroscopy can only detect paramagnetic species that contain unpaired electrons. Although this seems to be a limitation in comparison to other techniques, such as X-ray absorption and emission spectroscopy, as well as vibrational and electron spectroscopies, paramagnetic species are very frequently involved in catalytic cycles.^2^ For these species, EPR spectroscopy provides unique information on their electronic structure, since the measured magnetic properties of the unpaired electron(s) are directly linked to their electronic and geometrical properties.^2–5^ Therefore, the EPR spectrum of TMI-containing catalysts is highly sensitive to the oxidation state, ligand field and coordination geometry of the respective TMIs. Since these characteristics define the possible activity of the respective TMI centre, EPR spectroscopy provides a handle for determining the nature of the active species and unravelling reaction mechanisms. Lastly, since EPR also allows quantification of the sites with unpaired electrons, it is a suitable method for the reliable extraction of thermodynamic data.^3,6,7^

While time-resolved EPR has shown an enormous potential to gain molecular insights into working catalysts,^1,2,4,8,9^ the data acquired in the context of heterogeneous catalytic reactions often suffer from low signal-to-noise ratio (S/N) and/or poor time-resolution. In order to solve these challenges while simultaneously highlighting spectral features related to relevant species reversibly involved in the catalytic reaction, dynamic *operando* experiments can be performed according to the modulated excitation (ME) approach coupled with phase-sensitive detection (PSD) analysis.^10,11^ To demonstrate the feasibility of MES-PSD in combination with *operando* EPR spectroscopy and extract site-specific kinetic information we studied the CO-assisted N_2_O decomposition reaction on a commercial Fe–ferrierite (Fe–FER)^12^ catalyst.

Nitrous oxide (N_2_O) is the third most important greenhouse gas and since its anthropogenic emissions in nitric and adipic acid production plants and fossil fuel combustion units contribute to ozone layer depletion, effective catalytic after-treatment technologies are required. By possessing pronounced activity in N_2_O conversion strategies, Fe-exchanged zeolites are industrially implemented to control N_2_O emissions, generally *via* the decomposition route.^13^ In this view, upon an activation step (eqn (1)) in which N_2_O oxidizes a reduced active Fe centre (Fe^(*n*−1)+^) releasing N_2_, the rate of the reaction is controlled by the reduction half-cycle, *i.e.* the auto-reduction of the oxidized Fe centre (Fe^*n*+^–O) coupled with O_2_ formation (eqn (2)).^14^ Hence, reducing agents such as CO can be used to promote N_2_O conversion.^15^ Indeed, the overall reaction rate is promoted since CO accelerates the reduction half-cycle upon interaction with Fe^*n*+^–O (eqn (3)).^15,16^

1 Fe^(*n*−1)+^ + N_2_O → Fe^*n*+^–O + N_2_

2 2Fe^*n*+^–O → 2Fe^(*n*−1)+^ + O_2_

3 Fe^*n*+^–O + CO → Fe^(*n*−1)+^ + CO_2_

In the past decade, ground-breaking work from Solomon and co-workers lead to the elucidation of the active iron Fe^II^ species responsible for N_2_O activation, and the corresponding activation energies were reported based on DFT calculations.^17–19^ However, no site-specific kinetic data for the oxidation and reduction half-cycles (OHC and RHC, respectively) of the catalytically relevant Fe^II^ centres are available at realistic and operative temperatures (*i.e. T* > 300 °C).

According to the reactions in eqn (1) and (3), the active Fe centre/s are expected to undergo a red-ox process involving reversible Fe^II^/Fe^III^ red-ox. However, since only Fe^III^ species exhibit a half integer spin quantum number, only this oxidation state of the active centre/s is expected to exhibit a signal under our experimental conditions, whereas Fe^II^ and Fe^IV^ sites are non-detectable by EPR.^17^ Furthermore, EPR allows to differentiate between high spin (*S* = 5/2) and low spin (*S* = 1/2) Fe^III^ ions and rhombicity of the zero field splitting of the former is very sensitive to the distortion of the geometry of the respective site.^20^ The EPR spectrum of isolated high-spin Fe^III^ sites, commonly encountered in Fe-zeolites, is predominantly characterized by zero-field splitting (ZFS) contributions in the spin Hamiltonian. The observed transitions occur within different Kramer doublets, which are often approximated as fictitious *S* = 1/2 systems with an effective *g*′ value. In very high symmetries, such as cubic symmetry, the zero-field splitting vanishes to first order, typically resulting in signals around *g*′ = 2. Axial coordination produces characteristic signals at *g*′ = 6 and *g*′ = 2, while strong rhombic distortion results in signals at *g*′ = 4.3 and a long stretch up to *g*′ ≈ 9.^15,20–22^

Therefore, EPR is a highly suitable tool for differentiating Fe^III^ ions located in different cationic positions and determine their individual Fe^II/^Fe^III^ red-ox dynamics.

Given the reaction under investigation, we achieved ME *via* periodic variation of the gas composition, *i.e.* concentration of oxidizing (N_2_O) and reducing (CO) agents involved in the reaction (eqn (1) and (3)), while recording time-resolved EPR spectra to monitor the red-ox dynamic of the possibly active Fe centres. The raw time-resolved data collected during the pulse sequence can be averaged into one period, the so-called averaged time-resolved spectra, enhancing the S/N in function of the number of pulse sequences applied. In order to further emphasize the contribution of the active species the acquired data can be processed with a demodulation function (eqn (4)),^10,11^ which is equivalent to a digital lock-in amplifier suppressing the parts of the spectrum which are not affected by the modulation while maximizing the contribution to the spectra that were reversibly perturbed during the experiment.4
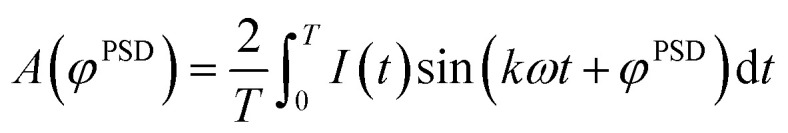


The transformation of time-resolved spectra consisting of intensity data (*I*(*t*)) into phase-resolved spectra is called phase sensitive detection (PSD). PSD is performed by multiplying the time-resolved spectra with a sine function of the same frequency as the stimulation (*ω*) and a demodulation phase angle (*φ*^PSD^), followed by integration over the modulation period (*T*). The resulting spectra contain only information on the signal/s of species that are reversibly perturbed during the experiment, *i.e.* which are modulated, and the time delay with which they respond to the external stimulus. Thus, in the phase-resolved spectra (*i.e.* demodulated spectra) the spectral contributions of the active species are maximized, simplifying their identification.

Despite the process of CO-assisted N_2_O decomposition possesses limited industrial applicability, we chose this model reaction because it develops cycling through an oxidation- and reduction half-cycle theoretically occurring at substantial rates and we wanted to prove that by combining *operando* EPR with MES and PSD, one can monitor a fast red-ox reaction without sacrificing spectral- or time-resolution.

Following this approach, it was possible to derive qualitative and quantitative information by isolating and identifying the active Fe centers responsible for the reversible red-ox cycle involving the oxidation of Fe^(*n*−1)+^ by N_2_O (eqn (2)) and the subsequent reduction of Fe^*n*+^ by CO (eqn (3)). The temporal evolution of these red-ox reactions was monitored by integrating the EPR transition corresponding to the active Fe centers and by using multivariate curve resolution (MCR). This approach allowed us to derive detailed kinetic information going beyond what can be typically achieved. The results show that MCR can produce reliable outputs in the framework of *operando* EPR spectroscopy.

## Materials and methods

A commercial Fe–FER catalyst provided by Casale SA, which has been previously fully characterized^12^ and possesses a Fe content of 0.7 wt% and a Si/Al ratio of 9.4 (from elemental analysis), was crushed and sieved to 100–150 μm for all EPR experiments (Fig. S1†).

**Scheme 1:**
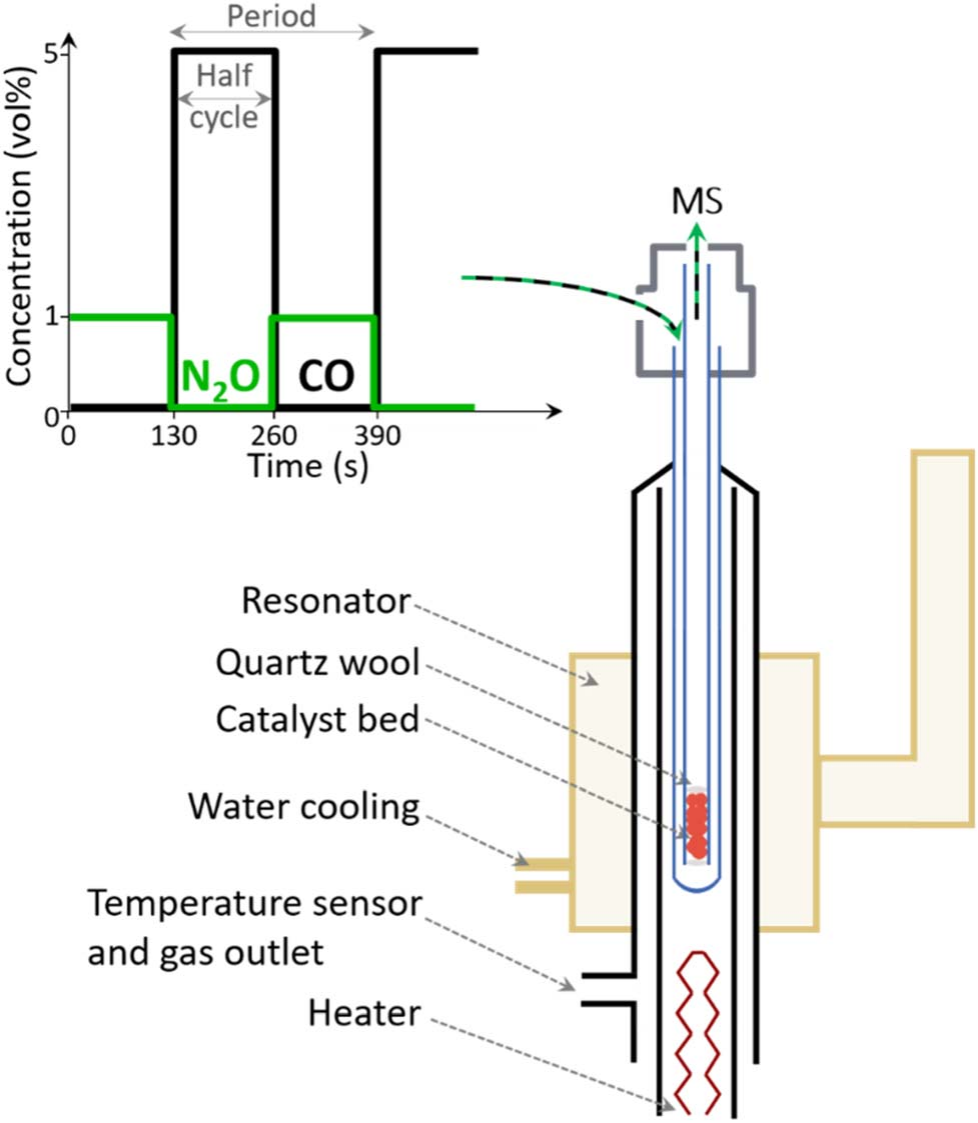
Schematic representation of the periodic variation of gas composition and high temperature setup used in modulation excitation experiments. The powder sample is exposed to alternated pulses of 1 vol% N_2_O/Ar and of 5 vol% CO/Ar in a constant flow of Ar. In each half-cycle (130 s) 10 spectra were recorded.

EPR measurements were performed using a homebuilt water-cooled high-temperature resonator which was plugged into a CW EPR spectrometer (Bruker EMX) operating at X-band frequencies (∼9.265 GHz).^23,24^ The resonator was heated by a flow of hot N_2_ gas controlled by a homebuilt temperature regulator. An EPR quartz tube (4 mm outer diameter – Wilmad Labglass) and quartz capillary (2 mm inner diameter – Qsil) were used as outer tube and inner capillary, respectively (Scheme 1).^25^ The material was fixed in the inner capillary between two quartz wool plugs (Roth). This assembly was connected to an experimental setup comprising mass flow controllers (Bronkhorst) and solenoid valves (Series 9, Parker) to control the gas flows and automatically switch between gases, respectively. A total flow rate of 15 ml min^−1^ and *ca.* 12 mg of catalyst were used for *in situ* and ME experiments.

*In situ* experiments were performed by heating Fe–FER in a flow of Ar to 673 K followed by exposure and equilibration of the catalyst first to 1 vol% N_2_O/Ar and then to 5 vol% CO/Ar at the same temperature.

ME experiments were performed by pulsing alternatively 1 vol% N_2_O/Ar and 5 vol% CO/Ar over Fe–FER equilibrated in Ar in the 573–673 K temperature range. Pulses were performed every 130 s for *ca.* 3 h (40 pulses) while recording a total of 20 spectra in one full period (1 spectrum = 13 s, see Table S1† for details). A mass spectrometer (Omnistar, Pfeiffer) connected downstream of the inner capillary was used for on-line monitoring of the outlet gas concentrations, *m*/*z* = 16 (CO_2_), 28 (CO), 40 (Ar) and 44 (N_2_O). In order to account for possible fluctuations in the total flow rate, after each experiment, the ion currents of the mass fragment of interest were normalized by the ion current of Ar, *i.e.* the inert gas. Since CO and N_2_O significantly contribute to *m*/*z* of 28 and 44, respectively, we chose to monitor CO_2_ following *m*/*z* = 16. The CO_2_ contribution in the gas composition was further extracted from the *m*/*z* = 16 signal taking into account that also CO contributes to the *m*/*z* signal. Furthermore, the ion current profile related to CO_2_ was smoothed using the Savitzky–Golay function built into the Origin 2021 software. The phase-resolved EPR spectra were obtained by averaging the time-resolved spectra from 40 modulation periods and by successively applying the PSD function (eqn (4)).^10,11,26^

Kinetic analysis on the temporal evolution of the Fe^II^/Fe^III^ transitions in Fe centres at *g*′ = 6.5 was performed after baseline subtraction of the averaged time-resolved EPR spectra by double integration in the region between 80 and 120 mT using Origin 2021 software. The spectrum at the end of the reduction half-cycle was used as an experimental baseline. The double integrated results were fitted using an exponential decay of the type *A*(*t*) = *A*_0_e^−*kt*^ + *C*, to derive kinetic constants (*k*) associated with the oxidation and reduction half-cycles employing the build in Levenberg–Marquardt-algorithm in the Origin 2021 software. Alternatively, the multivariate curve resolution with alternating least squares (MCR-ALS) approach was used by employing the Matlab toolbox MCR-ALS 2.0.^27^ Simulated EPR spectra calculated using the Matlab toolbox EasySpin^28^ (see Table S2†) were employed as initial guesses of the spectral components fitting the averaged time-resolved EPR spectra. Due to the derivative lineshape of the EPR data the non-negativity constraint could not be employed. In agreement with the simulations, component 2 was further restricted to the field range above 120 mT.

## Results and discussion

**Fig. 1:**
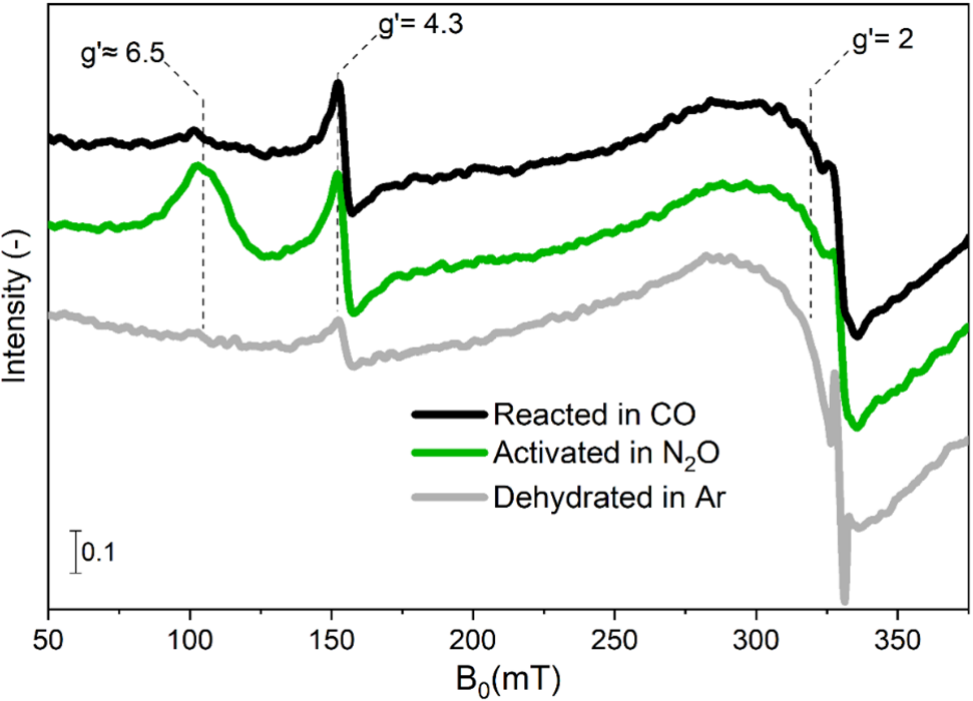
*In situ* EPR spectra of Fe–FER at 673 K after heating in Ar from RT to 673 K (bottom trace, grey), after oxidative treatment in 1 vol% N_2_O/Ar at 673 K (centre trace, green) and after reducing treatment in 5 vol% CO/Ar (top trace, black). The effective *g*′ values corresponding to transitions in different Fe centres are reported.

In a first attempt to investigate the red-ox dynamics during CO-assisted N_2_O decomposition, we performed *in situ* experiments in which Fe–FER was heated in Ar to 673 K and subsequently treated and equilibrated first in 1 vol% N_2_O/Ar and then in 5 vol% CO. The corresponding spectra are shown in Fig. 1.

After equilibration in N_2_O, the spectral features at effective *g*′ ≈ 6.5 and *g*′ = 4.3 increased in intensity in comparison to the spectrum of Fe–FER in Ar, while the transition resonating around *g*′ = 2 exhibited only a small increase in signal intensity. Due to the sensitivity of EPR spectroscopy regarding the oxidation state of transition metal ions,^17^ an increase in signal intensity can be associated with an oxidation process in which EPR-silent Fe^II^ centres initially present in Ar at 673 K are transformed in EPR-active Fe^III^ species.

The feature around *g*′ = 2 was previously assigned to highly symmetric isolated Fe^III^ species or small iron-oxide clusters in α position of the zeolite structure (10-membered rings).^14,29^ In this sample, the temperature dependence of the intensity of this feature did not follow the Curie–Weiss law, thus indicating that small iron-oxide clusters are the dominant species (Fig. S2†).^15^ The changes in intensity observed between room temperature and 673 K in the spectral region of 200–250 mT are attributed to the broadening of the Fe_*x*_O_*y*_ particles (Fig. S3†). The signal at *g*′ = 4.3 corresponds to isolated tetrahedrally-coordinated high-spin Fe^III^ sites in γ position, *i.e.* γ-Fe^III^ sites accommodated in a complex framework structure composed of 5-membered rings, based on the large rhombicity (*E* = *D*/3) of their zero field splitting (Table S2†).^14,30^ Finally, the signals at *g*′ ≈ 6.5 are assigned to isolated high-spin Fe^III^ sites in β position, (6-membered ring, β-Fe^III^).^22,29^ We recently demonstrated that in this Fe–FER catalyst such sites correspond to square pyramidal Fe^3+^ complexes.^31^

The N_2_O-treated catalyst was exposed to 5 vol% CO/Ar (Fig. 1) at the same temperature in order to investigate the RHC (eqn (3)). Comparison between the spectra for the oxidized and reduced state revealed that the signal associated with α-Fe^III^ and Fe_*x*_O_*y*_ clusters was largely static, suggesting that despite the partial ability to perform the OHC (eqn (1)), no remarkable Fe^III^/Fe^II^ red-ox behaviour can occur after switching the reaction atmosphere.^31^ Differently, the signal associated with β-Fe^III^ sites was almost completely consumed and the intensity related to γ-Fe^III^ species was only marginally affected after equilibration in CO-rich conditions. These results suggest that the β-Fe centres can provide full red-ox behaviour under reaction conditions, *i.e.* shuffle from EPR-silent β-Fe^II^ to EPR-active β-Fe^III^ and *vice versa*. However, these observations do not provide a definitive answer to whether also γ-Fe species can participate in both OHC and RHC (eqn (1) and (3), respectively).

In order to investigate this matter and explore the effective red-ox reversibility of the β-Fe and γ-Fe sites, dynamic *operando* experiments were performed by modulating the gas feed composition between 1 vol% N_2_O/Ar and 5 vol% CO/Ar at relevant temperatures (573 K ≤ *T* ≤ 673 K) while scanning the magnetic field in the region of interest (80–170 mT).

**Fig. 2:**
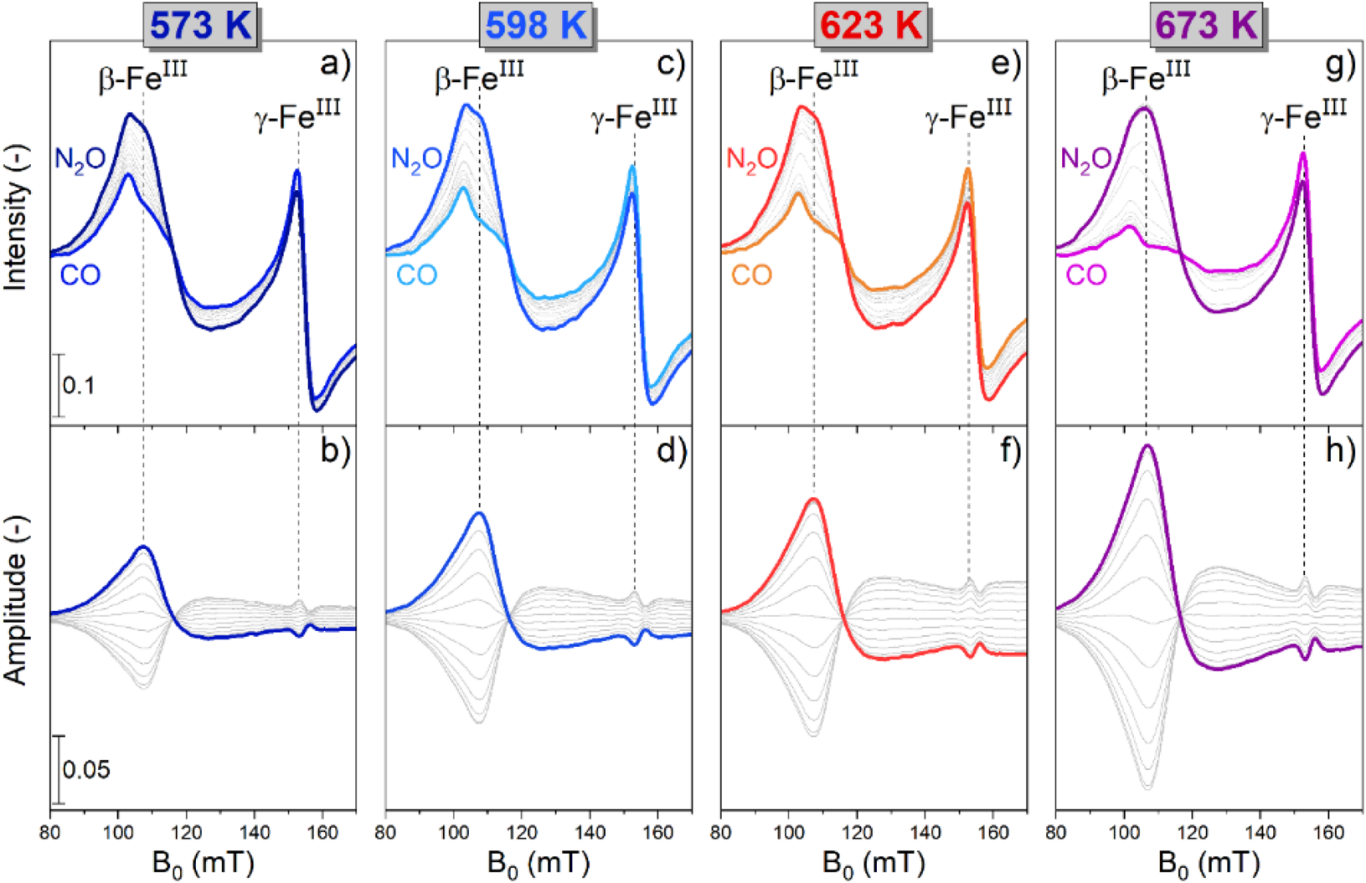
Averaged time-resolved (a), (c), (e) and (g) and phase-resolved (b), (d), (f) and (h) dynamic *operando* EPR spectra of Fe–FER during 130 s pulses of 1 vol% N_2_O/Ar alternated to 5 vol% CO/Ar in a constant flow of Ar at 573 K (a) and (b), 598 K (c) and (d), 623 K (e) and (f) and 673 K (g) and (h). The bold spectra in the time-resolved panels are drawn to indicate the effect of N_2_O or CO on the EPR spectra of the catalyst, while in the phase-resolved panels are drawn to guide the eye.

The raw time-resolved EPR spectra (Fig. S4†) displayed a reversible and periodic change in the intensity of the spectral features associated with β-Fe^III^ (107 mT) and γ-Fe^III^ (153 mT), thus demonstrating a potential red-ox dynamics, in agreement with previous findings.^31^ Simultaneously, a slight non-reversible trend was detected in the high field region of the spectra at 170 mT (Fig. S5†), which is associated with changes in the clusters and also in the signal associated to γ-Fe^III^ (Fig. S4h†), where a slow increase in intensity occurred over time at 673 K. *In situ* measurements demonstrated that the signal associated with the Fe_*x*_O_*y*_ clusters underwent a slow and irreversible change during the modulation experiment (Fig. S5 and S6†). This well-known behaviour is attributed to changes in size of the Fe_*x*_O_*y*_ clusters in CO-rich atmospheres.^15^ Hence, the drift in the signal intensity of γ-Fe^III^ at 673 K probably occurs because of the overlapping with the signal contribution related to the changing Fe_*x*_O_*y*_ clusters. Nevertheless, since the relevant transitions associated with β-Fe^III^ and γ-Fe^III^ display full reversible behaviour (Fig. S6†), the spectra collected during 40 modulation cycles were averaged and processed *via* PSD. This data treatment allows to maintain a sufficiently high time-resolution while simultaneously increasing the S/N thus assisting in monitoring fast chemical processes (Fig. S7†).^10^ The averaged time-resolved *operando* EPR spectra are reported in Fig. 2a, c, e and g.

The intensity of the signal associated with β-Fe^III^ centres increased in N_2_O-, and decreased in CO-rich environments irrespective of the reaction temperature. In agreement with the *in situ* experiments discussed previously (Fig. 1), β-Fe centres shuffled from the EPR-silent β-Fe^II^ state to the EPR-active β-Fe^III^ form when N_2_O was added, and *vice versa* upon introduction of CO in the reaction environment. In the case of the γ-Fe sites, the changes were subtle, which is in line with the data recorded in the *in situ* experiments (Fig. 1). Furthermore, given the overlapping between their signal and the transition associated with β-Fe^III^ centres, it is challenging to describe their behaviour just using these time-resolved results. Simultaneously with the spectroscopic changes, the online MS data (Fig. S8†) showed that the CO and CO_2_ signals were correlated, proving that CO_2_ was produced in the CO-rich part of the pulse sequence and that the red-ox dynamics observed by *operando* spectroscopy occurred while the CO-assisted N_2_O decomposition was proceeding according to eqn (1) and (3).

**Fig. 3:**
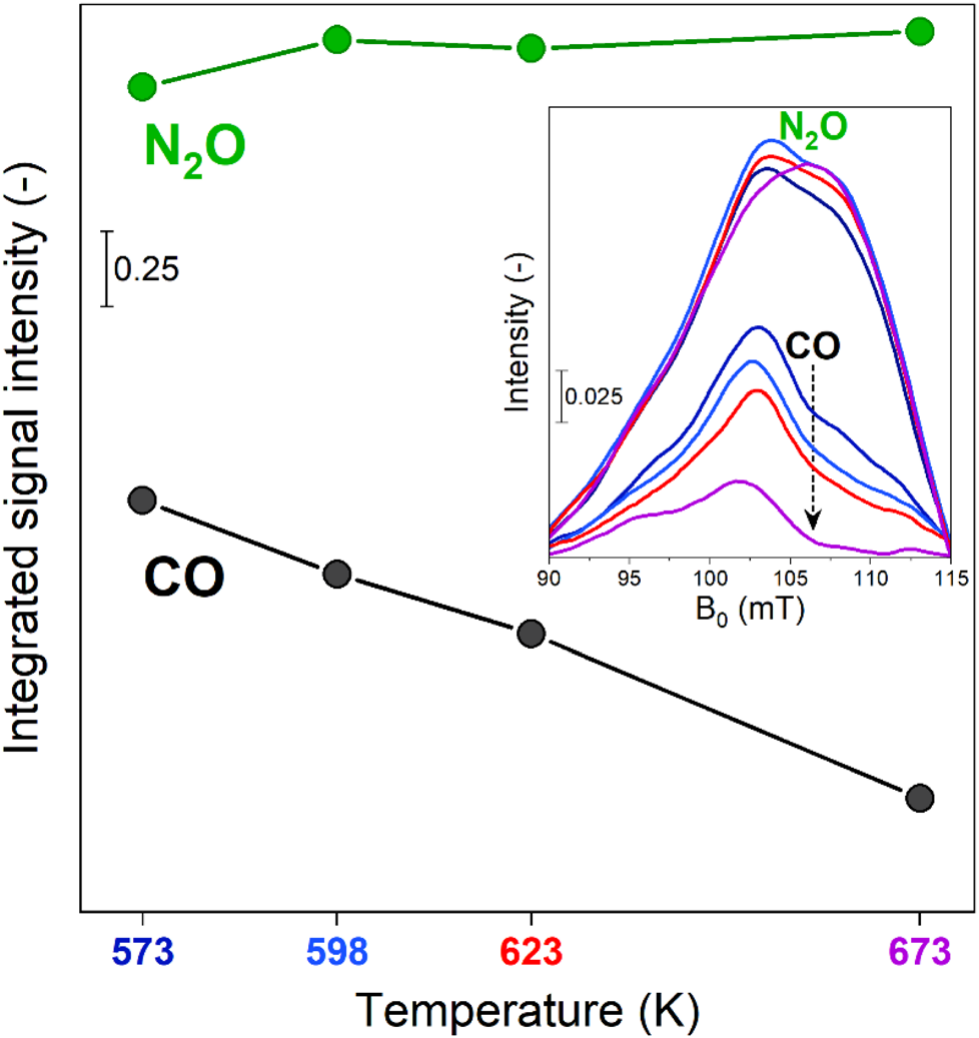
Integrated signal intensity of the spectral feature at *g*′ = 6.5 calculated from the spectra measured at the end of the N_2_O- or CO-half cycles at 573 K, 598 K, 623 K and 673 K (showed in the inset) during the time-resolved dynamic *operando* EPR experiments.

Comparison between the time-resolved EPR spectra (Fig. 2a, c, e and g) collected at different temperatures indicated that the most striking variation consisted in a progressive decrease in intensity of the β-Fe^III^ centres at the end of the CO-rich part of the pulse sequence. Differently, the intensity of their signal in the presence of N_2_O was only marginally affected by the temperature. In order to highlight this observation, the integrated signal intensity of the spectral feature associated with β-Fe^III^ sites at the end of the CO- or N_2_O-half cycles was plotted in Fig. 3. The data indicated a progressive decrease in the area of the signal at the end of the CO-half cycle, suggesting that CO can reduce a higher fraction of oxidized β-Fe^III^ centers during the half-cycle at higher temperature. Contrarily, the intensity of the β-Fe^III^ signal in N_2_O-rich environments was nearly constant, indicating that N_2_O can promote re-oxidation of all the available β-Fe^II^ centres irrespective of the reaction temperature, thus indicating that the re-oxidation of the EPR-silent β-Fe^II^ centres is probably not rate-limiting. Since the fraction of oxidized β-Fe^III^ sites in oxidizing conditions is almost constant while the extent of reduction in CO-rich conditions progressively increases at higher temperature, the RHC is probably rate-limiting.

The modest change in the line shape of β-Fe^III^ in the oxidation and reduction half cycle visible in Fig. 2 could point towards the presence of several species with slightly different ZFS due to variation in the local Al^III^ distribution or small changes in the ZFS in the different chemical environments in the RHC and OHC.

**Fig. 4:**
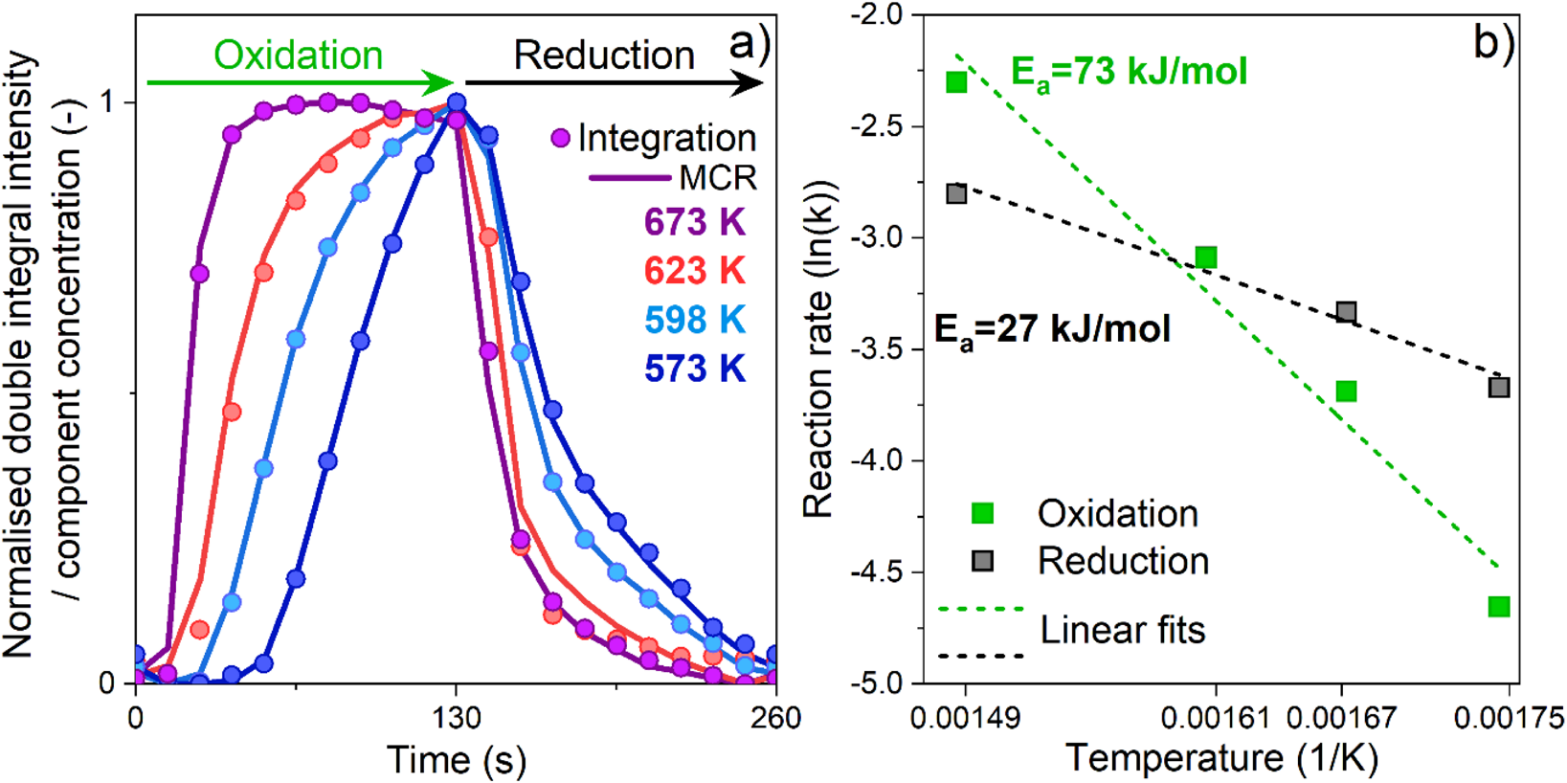
Temporal evolution of the spectral feature at *g*′ = 6.5 during the oxidation and reduction half-cycles (a) expressed as (i) normalized intensity of the double integrated EPR results (dots) and concentration profiles obtained by MCR analysis (line). Arrhenius plots for the reduction and oxidation half-cycles (b) using kinetic constants derived from the time-resolved *operando* EPR spectra (see Experimental section, Table S3 and Fig. S13†).

The phase-resolved results (Fig. 2b, d, f and h) constructed by application of the PSD function (eqn (4)) clearly emphasize that the majority of the reversible changes in the time-resolved spectra were associated with the β-Fe sites. However, also the signal associated to transitions in γ-Fe^III^ centres appeared in the demodulated spectra, indicating that they also responded to the applied perturbation. Considering the β-Fe sites, the observation of demodulated signal within the whole temperature range investigated demonstrates that they are reversibly perturbed, and thus ensure reversible red-ox dynamics, irrespective of reaction temperature. With the timing of half cycles that we use, the extent of this reversible variation increases at higher temperatures, primarily because the reduction – probably the oxidation as well – does not proceed to completion within each half cycle, as indicated in Fig. 4a. This observation demonstrates that the fraction of β-Fe sites shuffling according to reversible Fe^II^/Fe^III^ transitions grows at higher temperature, in agreement with the more pronounced reduction shown above (Fig. 3).

During these *operando* EPR experiments the signals related to the γ-Fe^III^ and β-Fe^III^ sites (*g*′ = 4.3 and *g*′ ≈ 6.5, respectively) were in anti-phase (Fig. S9†), *i.e.* when the signal intensity of γ-Fe^III^ increased, the one associated with β-Fe^III^ decreased. Accordingly, since the intensity of the γ-Fe^III^ feature decreased when N_2_O was introduced and increased in CO-rich atmosphere, the associated dynamics cannot originate from changes in valence state while participating in the reactions reported in eqn (1) and (3). The small increase in intensity can be attributed to a decrease in the linewidth of the γ-Fe^III^ species. One possible explanation could be a slight change in geometry in CO-rich atmosphere, which in turn changes the zero-field splitting. Alternatively, the dipole–dipole interaction may decrease due to the reduction of a fraction of the β-Fe^III^ sites to the β-Fe^II^ form.^32^ Irrespectively, the only active species able to undergo the full red-ox dynamics required for the CO-assisted N_2_O decomposition under these conditions are the β-Fe sites, which, exploiting the PSD paradigm, can be evidently recognized and isolated from the inactive species.

Since our results demonstrate that β-Fe centres are the only active species, the temporal evolution associated with their red-ox dynamics was investigated. On the one side, we double integrated their EPR signal intensity in the interval of 80–120 mT (Fig. S10†), and simultaneously, the averaged time-resolved spectra were analysed with a multivariate curve resolution algorithm (MCR)^27^ assuming two different components (see Fig. S11, S12 and Table S2†). The concentration profiles of the β-Fe^III^ sites obtained with both methods are shown in Fig. 4.

Irrespective of the approach, the results are in good agreement with each other, proving that the MCR method can provide reliable and useful information for analysis of EPR spectra and unravel kinetic trends of its components. The temporal evolution of the β-Fe^III^ sites exhibited different profiles in the OHC compared to the RHC. In the former case, the time-profile indicated the presence of two regimes upon N_2_O introduction. A first induction period, in which the intensity of the signal did not increase, was followed by a regime in which the signal intensity grew. This suggests that in the OHC under our experimental conditions an additional step precedes the actual oxidation of the EPR-silent β-Fe^II^ centres by N_2_O. Furthermore (i) the length of the induction period progressively decreased and (ii) the effective oxidation profile became steeper, with increasing temperature. It has been shown that CO and CO_2_ can adsorb over Fe^II^ species at room temperature.^33^ Therefore, we speculate that CO is adsorbed over the β-Fe^II^ centres after the RHC during the CO-rich part of the experiment, and needs to be removed by N_2_O before the actual re-oxidation of the catalytically relevant EPR-silent β-Fe^II^ centre can occur. Alternatively, N_2_O molecules might require rearrangement after the adsorption on Fe^II^ and prior to the O–N_2_ bond cleavage and Fe^II^/Fe^III^ transition.^18^ However, a definitive and more detailed chemical interpretation is beyond the scope of this paper.

To gain further insights into the reaction dynamics, the oxidation and reduction profiles derived from the integration were fitted with a first-order reaction rate law, neglecting the points collected during the induction periods characterizing the OHC (Fig. S13†). The kinetic constants derived from the fitting are reported in Table S3† and were used for construction of the Arrhenius plot of Fig. 4b. Linear fitting of these data allowed to extract the apparent activation energies. For the OHC, an activation energy of 73 (±9) kJ mol^−1^ was found, whereas the activation energy of the RHC was substantially lower (about 27 ± 4 kJ mol^−1^). The decomposition of N_2_O resulting from the oxidation of Fe^II^ to Fe^III^ has been studied both experimentally and theoretically. Values of 53.5 kJ mol^−1^,^18^ 68 kJ mol^−1^ (ref. 16 and 34) and 81–85 kJ mol^−1^ (ref. 35) have been reported, showing an overall good agreement with the value found in this work. Differently, for the CO assisted decomposition of Fe–O (eqn (3)) experimental values in the range of 39–50 kJ mol^−1^ and theoretical predictions of 56 kJ mol^−1^ can be found for Fe–BEA.^16^

Our results indicate a lower apparent activation energy for the reduction of Fe^III^ in CO rich atmosphere. This discrepancy with the value derived by us probably originates from the different Fe speciation and content as well as different experimental conditions used in the studies.

At a first grasp, these results might indicate that the OHC controls the overall reaction rate because of its higher apparent activation energy. However, these kinetic results derive from analysis of the normalized temporal evolution of the Fe^II^/Fe^III^ red-ox (and *vice versa*) in the active β-Fe sites within the reversible regime. They do not take into consideration that the average degree of oxidation and reduction, and thus the extent of reversibility, might change and shift towards one of the two conditions depending on the reaction temperature. In fact, we showed that the extent of reversible Fe^II^/Fe^III^ transitions does change (Fig. 3). While the degree of oxidation is constant in the temperature regime investigated and N_2_O can fully oxidize the available β-Fe^II^ sites irrespective of temperature, the degree of Fe^III^/Fe^II^ transition at the end of the RHC in CO-rich conditions is highly temperature-dependent and, thus, limits the process. Accordingly, the different values in apparent activation energies herein reported give an indication of how strongly the temperature affects the rates of the oxidation and of the reduction reactions. They do not take in consideration the extent of oxidation and reduction achieved. In the case of the OHC, an increase in temperature does not lead to an increase in the fraction of active Fe^II^ centers that N_2_O can oxidize in the half-cycle. However, it strongly increases the rate at which their complete oxidation is achieved. Differently, in the case of the RHC, an increase in temperature does increase both the fraction of Fe^III^ centres that is reduced to the Fe^II^ state in the half-cycle and the rate of this reduction reaction in CO-rich conditions.

## Conclusion

In this study, the potential of combining *operando* EPR spectroscopy with modulation excitation (ME) and phase sensitive detection (PSD) methodologies for time-resolved monitoring of red-ox mediated catalyzed reactions was investigated. Focusing on the CO-assisted N_2_O decomposition and using a commercial Fe–FER catalyst, we demonstrated that isolated Fe^II^ centers in β-cationic positions are the only active sites sustaining the reaction by undergoing reversible Fe^II^/Fe^III^ transitions. Isolated Fe^II^ sites in γ-cationic positions and oligomeric Fe species are spectator species during the reaction since they can ensure only the oxidation half-cycle. Being stacked in the oxidized state, they do not exhibit the reversible red-ox dynamics required for the red-ox mediated reaction under study. Following the temporal evolution of the reversible β-Fe^II^/Fe^III^ transitions, it has been shown that the concentration profiles obtained from double integration of the EPR results and from multivariate curve resolution analysis (MCR) are in excellent agreement with each other, thus proving that MCR analysis can be applied in the framework of EPR spectroscopy. The kinetic analysis of these profiles indicated that an increase in temperature promotes more the rate of conversion from β-Fe^II^ to β-Fe^III^ in N_2_O-rich conditions compared to the reduction of β-Fe^III^ to β-Fe^II^ in the presence of CO. However, we would like to stress that despite the kinetic analysis of these reversible red-ox transitions yielded important information, one should also always take into account the extent of these processes, *i.e.* the degree of oxidation and reduction, in order to deliver reliable information on the half-cycle limiting the red-ox mediated reaction.

The implementation of MES-PSD in EPR spectroscopy provides a versatile experimental toolbox for monitoring and differentiating catalytically active species. This methodology is certainly transferable to other metal centers such as Cu^II^, V^IV^, and Mn^II^, as well as applicable with other stimuli such as temperature and pressure modulation. As demonstrated, MES-PSD is particularly effective at disentangling active centers from observer species and differentiating between individual kinetics of various active sites. Current experimental efforts aim to explore the limitations of this approach in respect to the number of different species. In some systems, spin-lattice relaxation, especially at high temperatures, can be quite fast, resulting in very broad lines. MES-PSD can help elucidate subtle changes in broad lines that would otherwise be indistinguishable from background noise. Furthermore, this approach extends to other fields of catalysis where different stimuli can be applied, such as periodic perturbations with electric potential in electrocatalytic studies or the modulation of incident light beams in photocatalysis. These techniques have the potential to enhance time resolution and improve SNR, thus enabling the detection of short-lived intermediate species.

## Data availability

All data that support the plots within this manuscript and other findings of this study are available from the corresponding authors upon request.

## Author contributions

Jörg W. A. Fischer: conceptualization, methodology, formal analysis, investigation, data curation, visualization, writing – original draft, writing – review & editing. Filippo Buttignol: conceptualization, methodology, formal analysis, investigation, data curation, visualization, writing – original draft, writing – review & editing. Alberto Garbujo: resources, writing – review & editing, supervision, project administration, funding acquisition. Davide Ferri: conceptualization, validation, data curation, resources, writing – review & editing, supervision, project administration, funding acquisition. Gunnar Jeschke: conceptualization, validation, data curation, writing – review & editing, supervision, project administration, funding acquisition.

## Conflicts of interest

The authors declare no competing financial interest.

## Supplementary Material

SC-016-D4SC07195F-s001
