# Supplementary material for: Elucidation of site-specific red-ox kinetics in the CO-assisted N2O decomposition over Fe–ferrierite by combining modulation excitation with operando EPR spectroscopy
Source: Chem Sci. 2025 Feb 4;16(11):4884–91. doi: 10.1039/d4sc07195f (PMC11808333; doi:10.1039/d4sc07195f)
Supplement: SC-016-D4SC07195F-s001 [file SC-016-D4SC07195F-s001.pdf]

Supplementary Information (SI) for Chemical Science.
This journal is © The Royal Society of Chemistry 2025

Supplementary information

Elucidation of site-specific redox kinetics in CO-assisted N_2O decomposition over Fe-Ferrierite by combining modulation excitation with *operando* EPR spectroscopy

Jörg W.A. Fischer[a,†], Filippo Buttignol[b,†], Alberto Garbujo[c], Davide Ferri[b,*], Gunnar Jeschke[a,*]

[a]*Department of Chemistry and Applied Biosciences, ETH Zurich, CH-8093 Zurich, Switzerland*

[b]*Paul Scherrer Institut (PSI), CH-5232 Villigen, Switzerland*

[c]*Basic Research Department, Casale SA, CH-6900 Lugano, Switzerland*

[†]These authors have contributed equally

* Corresponding authors: davide.ferri@psi.ch and gunnar.jeschke@phys.chem.ethz.ch

Table of contents

Supplementary Tables……………………………………………………………………………2

Supplementary Figures…………………………………………………………………………...5

Supplementary References……………………………………………………………………...18

Table S1

Measurement parameters of the EPR experiments.

Experiment	Sweep width (mT)	Modulation frequency (kHz)	Modulation amplitude (mT)	Sweep time (s)	Conversion time (ms)	Time constant (ms)	Power attenuation (dB)
ME	90	100	0.2	13	20	20	16
In situ	400	100	0.2	240	40	40	16

Table S2

Simulation parameters for the isolated Fe^{3+} species located in γ- (g' = 4.3) and β- (g' = 6.5) cationic positions. All simulations were performed with the Matlab package EasySpin.[1]

**Effective *g*' value**	***g*' = 4.3**	***g*' = 6.5**
g	2.0023	2.0023
Linewidth (mT)	6	10
E/*D*	0.33	0.02

Typically, for high-spin Fe^{III} sites, the g-tensor is assumed to be approximately isotropic and close to the free-electron value, $g \approx g_e$. Hence for simplicity in all line shape calculations, *g* was set equal to 2.0023.[2] The zero-field splitting in high spin Fe^{III} species is often much larger than the electron Zeeman interaction hence the effective *g*' value (the resonance position) is determined by the ratio of *E*/*D*. The absolute values of *D* and *E* can therefore not be determined.[3]

Table S3

Kinetic constants for the oxidation and reduction half-cycles extracted from the first-order fits* shown in Figure S9.

**Temperature (K)**	**Kinetic constant of oxidation (s^{-1})**	**Kinetic constant of reduction (s^{-1})**
573	0.00952	0.02545
598	0.025	0.03571
623	0.04545	0.04566
673	0.1	0.06061

*Fit equation: $A(\mathrm{t})=A_0\ \mathrm{e}^{-k*t}+C$

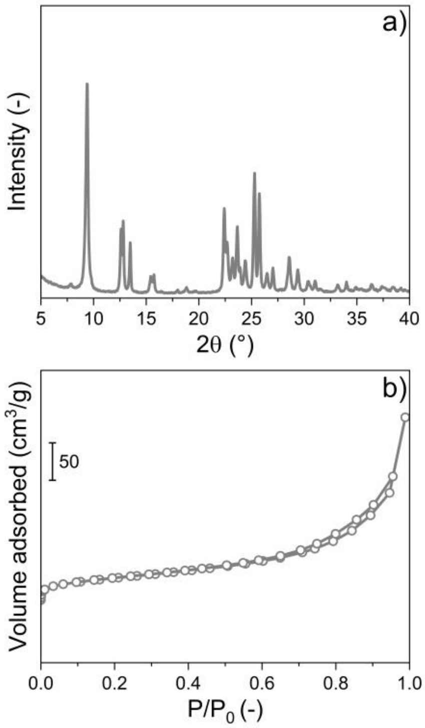

Figure S1: (a) XRD pattern and (b) N2 adsorption–desorption isotherms Fe-FER. Data shown for completeness and reproduced from [4]

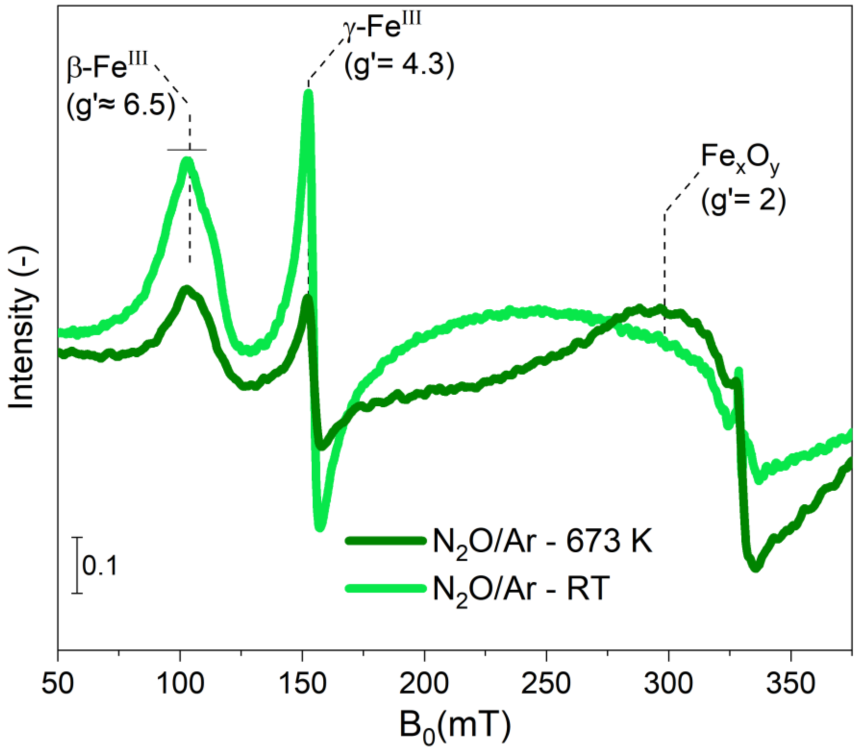

Figure S2: *In situ* EPR spectra of Fe-FER recorded at i) 673 K and ii) at room temperature in 1 vol% N_2O/Ar. The increased signal intensity in the transition at *g*' = 2 indicates that small ferromagnetic iron-oxide clusters are the dominant species.

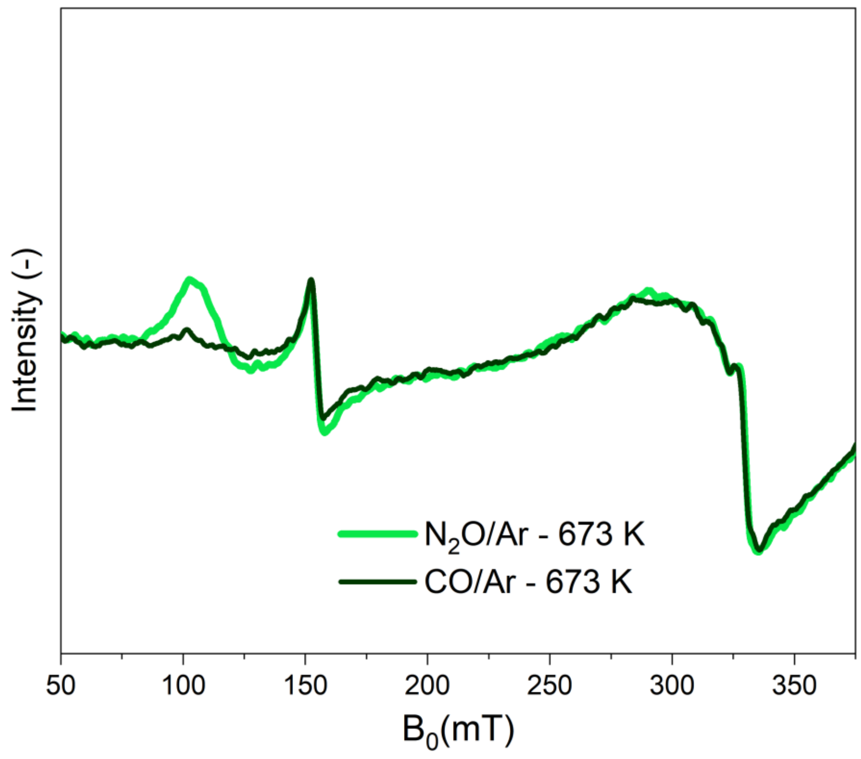

Figure S3: *In situ* EPR spectra of Fe-FER recorded at i) 673 K in 1 vol% N_2O/Ar and ii) 673 K in 5 vol% CO/Ar.

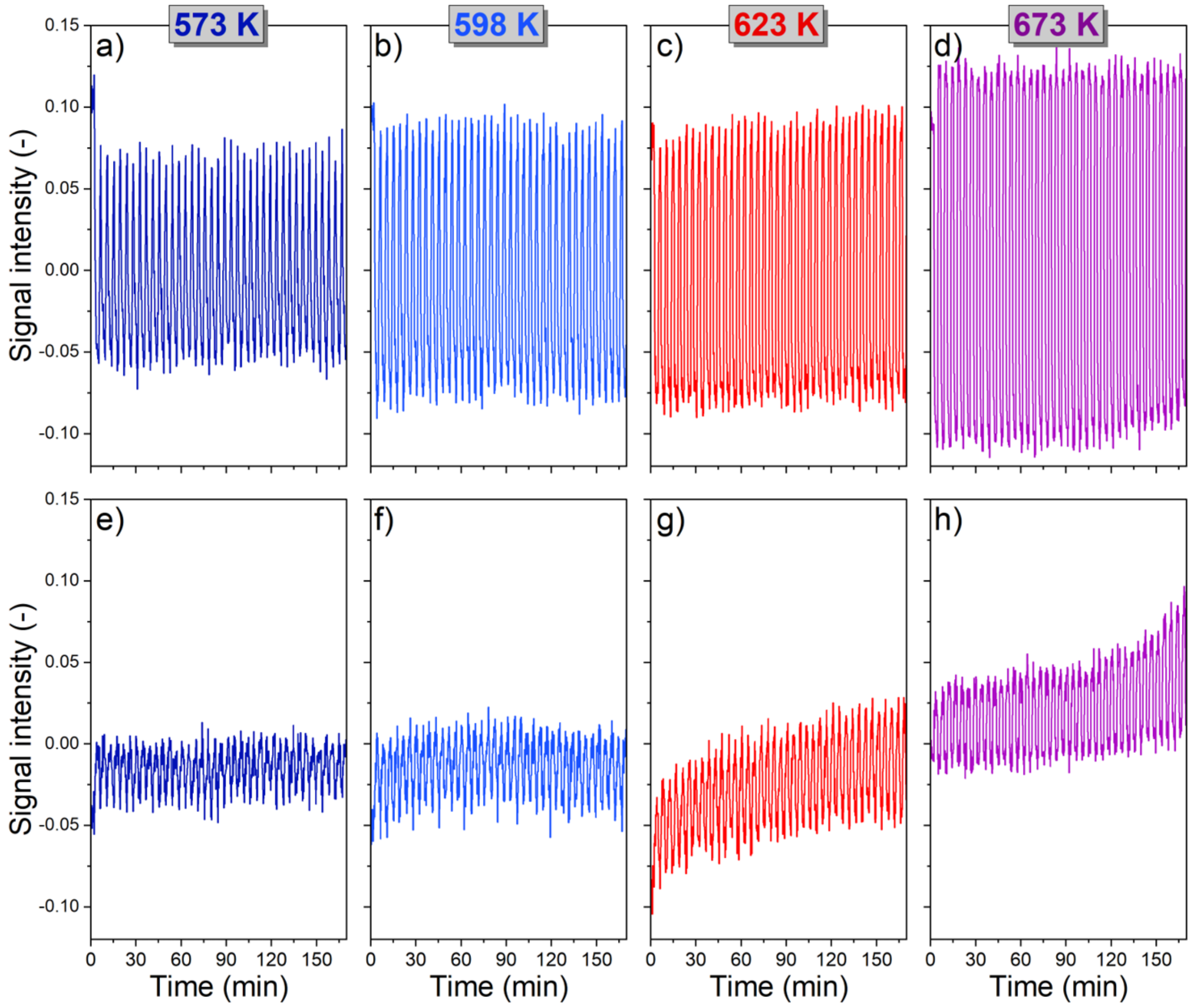

Figure S4: Signal intensity of the spectral features resonating at g’ ≈ 6.5 (a, b, c, d) and g’ = 4.3 (e, f, g, h) in Fe-FER monitored during ME-EPR experiments at 573 K (a, e), 598 K (b, f), 623 K (c, g) and 673 K (d, h).

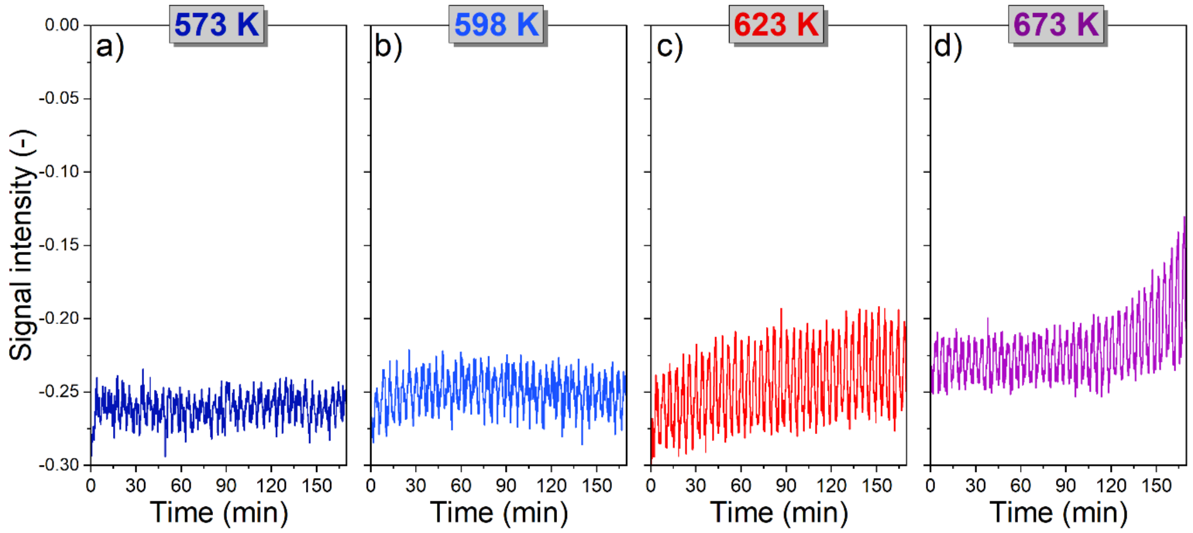

Figure S5: Signal intensity of the EPR spectra at 170 mT (g ≈ 3.8) in Fe-FER monitored during ME-EPR experiments at 573 K (a), 598 K (b), 623 K (c) and 673 K (d).

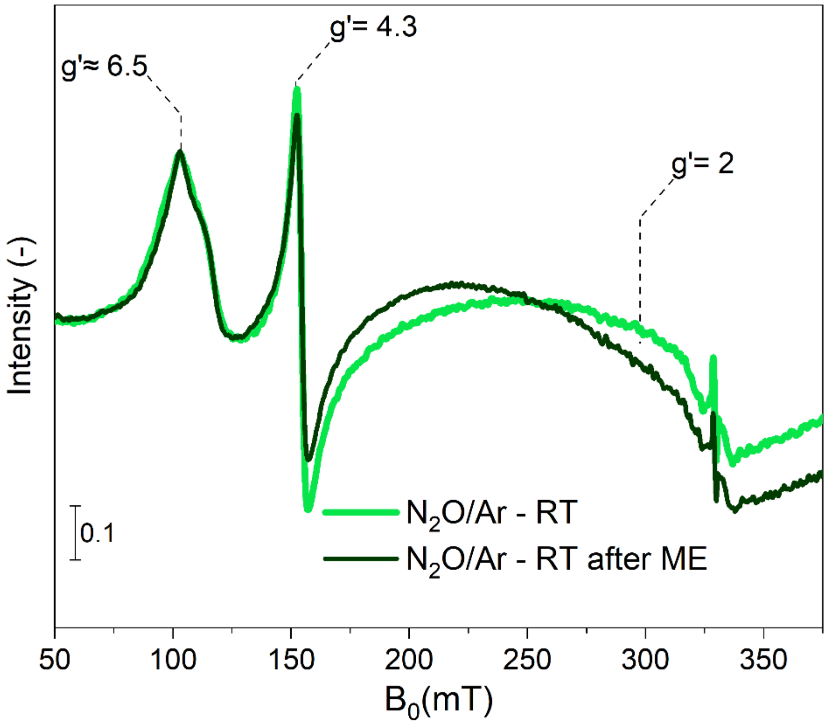

Figure S6: *In situ* EPR spectra of Fe-FER at room temperature in 1000 ppm N_2O/Ar i) before or ii) after the ME-EPR experiments. While full reversibility is observed for the transitions at g' ≈ 6.5, the signal in the region between 150 – 400 mT is evidently altered.

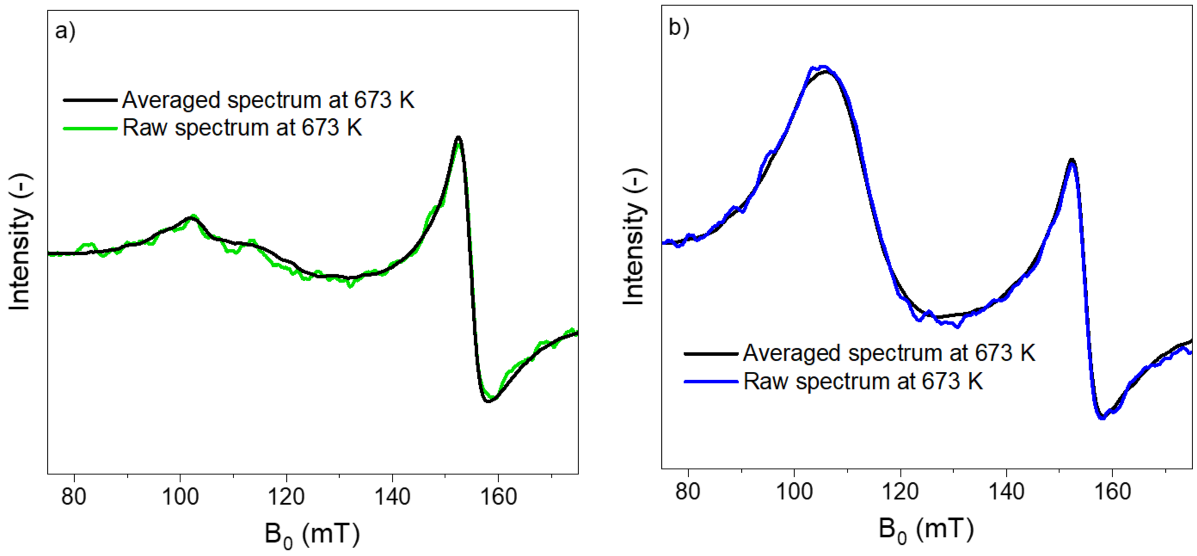

Figure S7: *Operando* EPR spectra of Fe-FER in a) the reduction half cycle and b) oxidation half cycle. The colored spectra are raw spectra before averaging, whereas the spectra in black are the averaged spectra at the same point in the respective reaction half cycle. The gain in SNR due to the averaging is clearly visible.

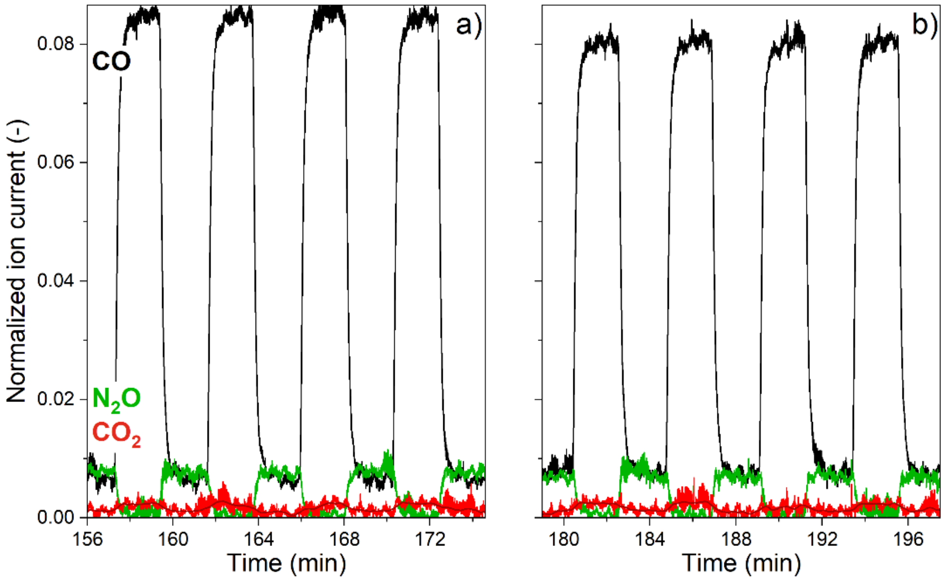

Figure S8: On-line MS signals during repeated pulses between 1 vol% N_2O/Ar and 5 vol% CO/Ar in a constant flow of Ar at 573 K (a) and 673 K (b) while recording *operando* EPR spectra.

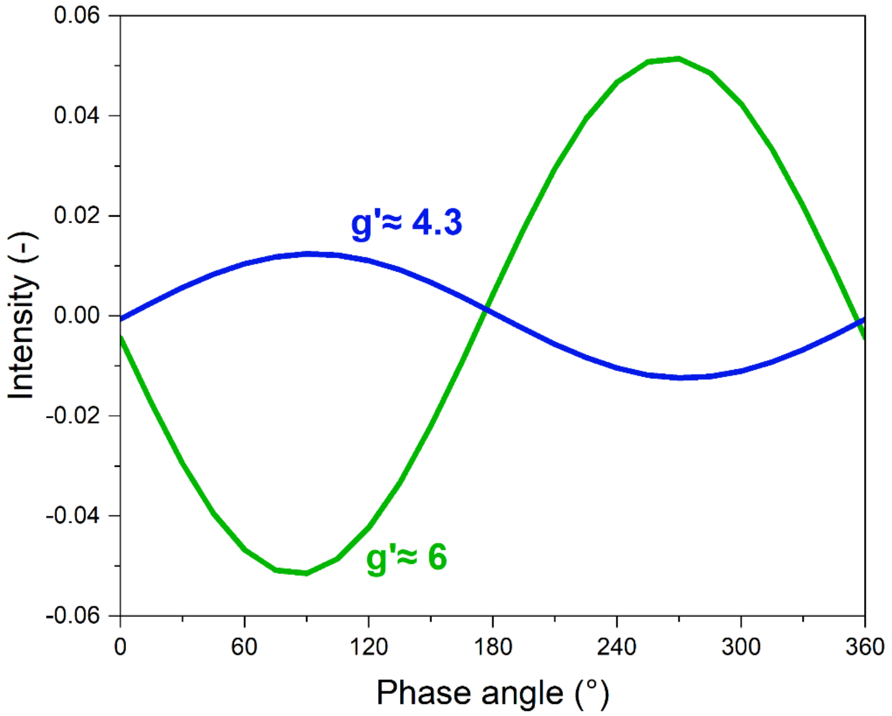

Figure S9: Phase-angular intensity variation for the spectral features at g'≈ 6 and at g' = 4.3. The maximum of the feature at g' = 4.3 is at the same phase angle of the minimum of g'≈ 6. Hence, both are in anti-phase.

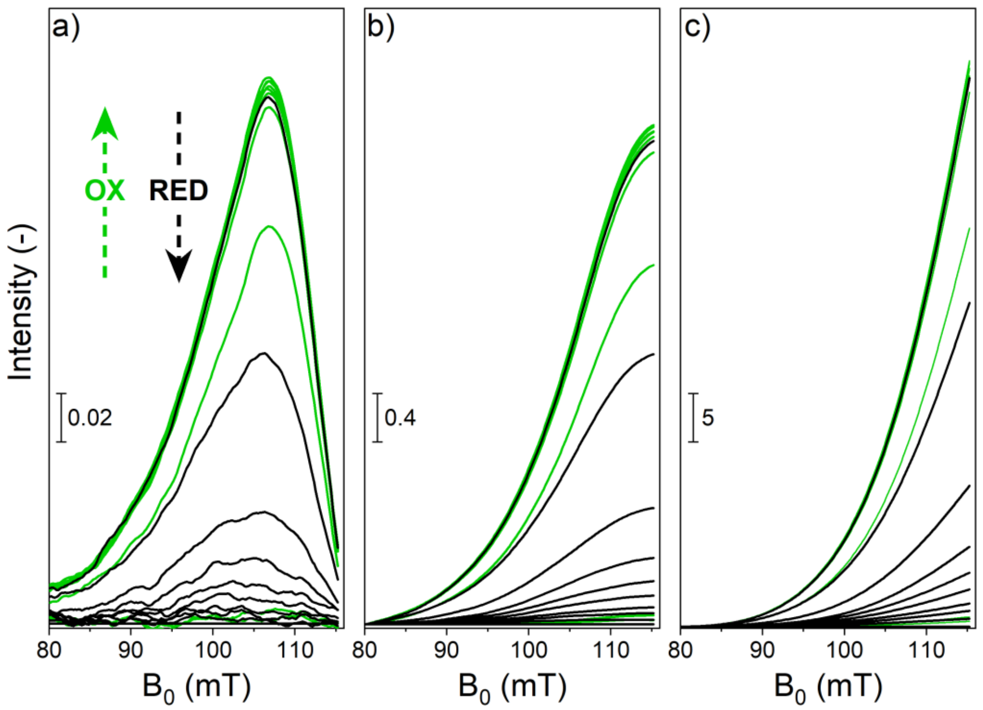

Figure S10: Schematic description of the integration process of the transition at g’≈ 6.5 from the averaged time-resolved EPR spectra during modulation excitation experiment at 673 K. Baseline corrected spectra of the full red-ox cycle (a), first integration of the spectra (b) and double integration of the data (c). The variation of intensity in the double integrated results at 116 mT was used to construct the concentration profiles of Figure S10.

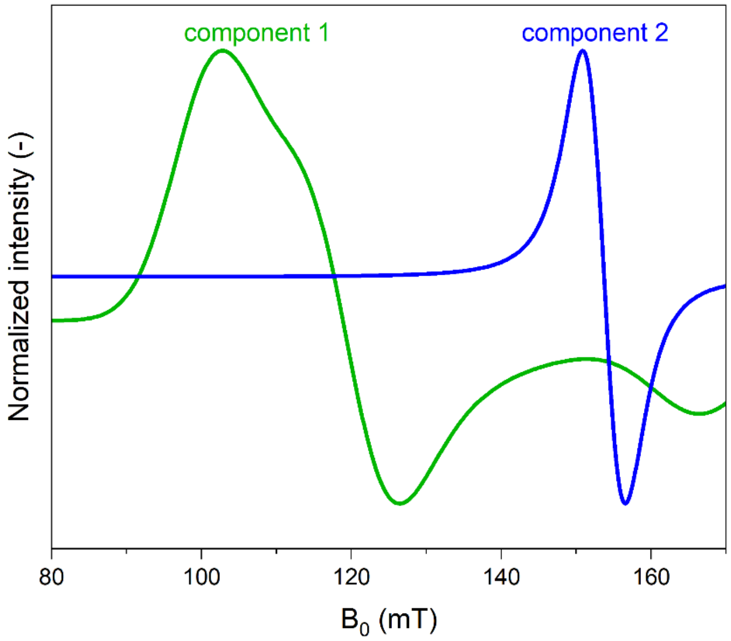

Figure S11: Initial guess of the spectral components used for MCR analysis of the averaged time-resolved *operando* EPR spectra.[5] The initial guess are simulated EPR spectra of the room temperature *in situ* spectrum (Fig. S2) which were calculated using the Matlab toolbox EasySpin[1] (see Table S2).

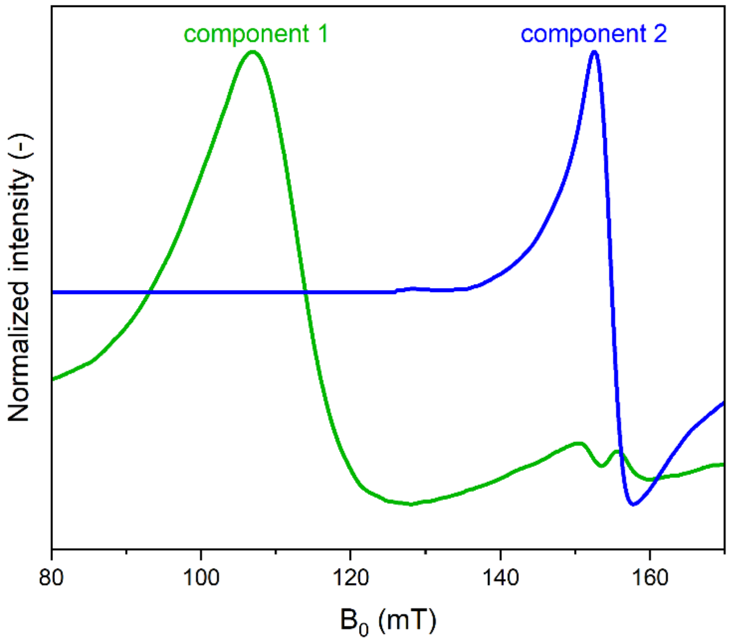

Figure S12: Spectral components obtained from MCR analysis of the averaged time-resolved *operando* EPR spectra.

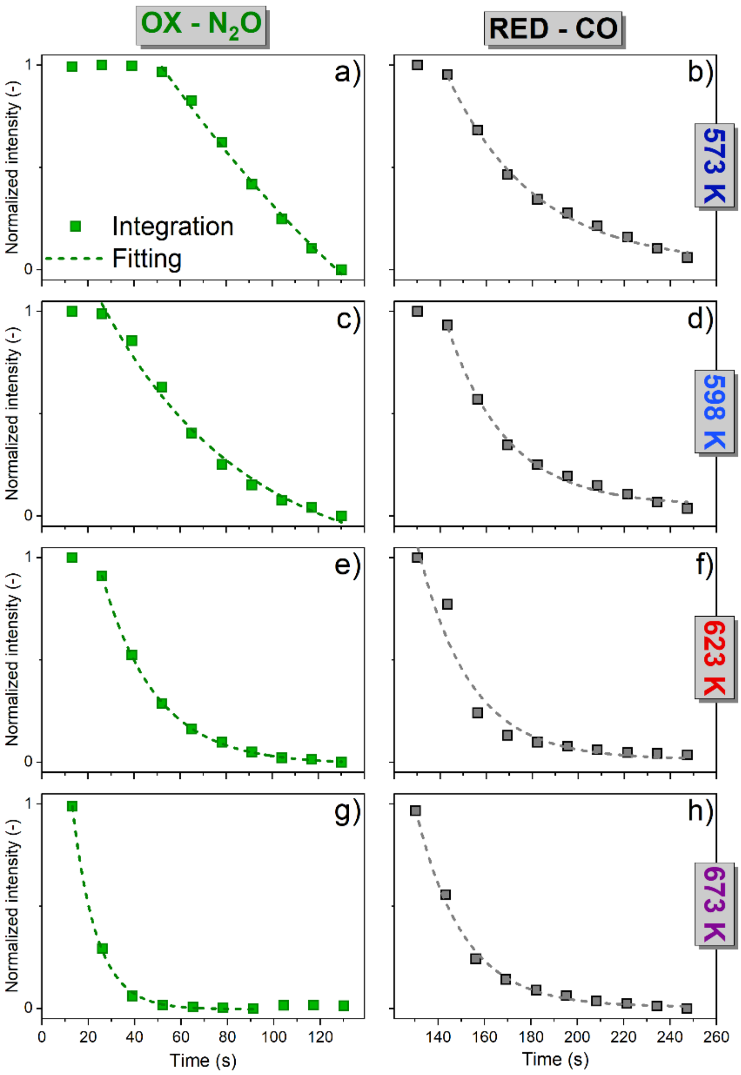

Figure S13: Normalized intensity profile of the double integrated data calculated from the averaged time-resolved *dynamic* operando EPR spectra during the oxidation half- cycles in 1 vol% N_2O/Ar (a, c, e, g) and during the reduction half-cycle in 5 vol% CO/Ar (b, d, f, h) at 573 K (a, b), 598 K (c, d), 623 K (e,f) and 673 K (g, h). The results of the fitting employing a first order reaction law, i.e. $A(t)=A_0 \cdot e^{-kt} + C$, are also reported. Depending on the experiment, the window in which fitting of the double integrated data has been performed is different.

References

1 S. Stoll and A. Schweiger, *J. Magn. Reson.*, 2006, **178**, 42–55.
2 R. Aasa, *J. Chem. Phys.*, 2003, **52**, 3919–3930.
3 T. Castner Jr., G. S. Newell, W. C. Holton and C. P. Slichter, *J. Chem. Phys.*, 2004, **32**, 668–673.
4 F. Buttignol, D. Rentsch, I. Alxneit, A. Garbujo, P. Biasi, O. Kröcher and D. Ferri, *Catal. Sci. Technol.*, 2022, **12**, 7308–7321.
5 J. Jaumot, A. de Juan and R. Tauler, *Chemom. Intell. Lab. Syst.*, 2015, **140**, 1–12.
